# Thoracic Ultrasound as an Alternative to Chest X-ray in Thoracic Surgery Patients: A Single-Center Experience

**DOI:** 10.3390/jcm13133663

**Published:** 2024-06-23

**Authors:** Luigi Lione, Alberto Busetto, Vincenzo Verzeletti, Giorgio Cannone, Alessandro Bonis, Alessandro Berni, Daniele Gasparini, Marco Mammana, Alessandro Rebusso, Samuele Nicotra, Dario Gregori, Andrea Dell’Amore, Federico Rea

**Affiliations:** 1 Thoracic Surgery Unit, Department of Cardiac, Thoracic and Vascular Sciences and Public Health, University of Padova, Via N. Giustiniani, 2, 35121 Padova, Italy; alberto.busetto@aopd.veneto.it (A.B.); vverzeletti@gmail.com (V.V.); giorgio.cannone@phd.unipd.it (G.C.); alessandro.bonis@aopd.veneto.it (A.B.); alessandro.berni@aopd.veneto.it (A.B.); marco.mammana@aopd.veneto.it (M.M.); alessandro.rebusso@aopd.veneto.it (A.R.); samuele.nicotra@aopd.veneto.it (S.N.); federico.rea@unipd.it (F.R.); 2 Unit of Biostatistics, Epidemiology and Public Health, University of Padua, Via L. Loredan 18, 35131 Padova, Italy; daniele.gasparini@ubep.unipd.it (D.G.); dario.gregori@ubep.unipd.it (D.G.)

**Keywords:** lung ultrasound, thoracic surgery, postoperative, clinical management

## Abstract

**Background/Objectives**: Chest X-ray (CXR) is currently the most used investigation for clinical follow-up after major noncardiac thoracic surgery. This study explores the use of lung ultrasound (LUS) as an alternative to CXR in the postoperative management of patients who undergo major thoracic procedures. **Methods**: The patients in our cohort were monitored with both a CXR and a lung ultrasonography after surgery and the day after chest drain removal. The LUS was performed by a member of the medical staff of our unit who was blinded to both the images and the radiologist’s report of the CXR. Findings were compared between the two methods. **Results**: In the immediate postoperative evaluation, 280 patients were compared, finding general agreement between the two procedures at 84% (kappa statistic, 0.603). The LUS showed a sensibility of 84.1%, a specificity of 84.3%, a positive predictive value (PPV) of 60.9%, and a negative predictive value (NPV) of 94.8%. We evaluated 219 out of 280 patients in the postdrainage-removal setting due to technical issues. Concordance between the methods in the postdrainage-removal setting was 89% (kappa statistic, 0.761) with the LUS demonstrating an 82.2% sensibility, a 93.2% specificity, a PPV of 85.7%, and an NPV of 91.3%. **Conclusions**: The results of this study showed a substantial agreement between LUS and CXR, suggesting that the LUS could reduce the number of X rays in certain conditions. The high NPV allows for the exclusion of PNX and pleural effusion without the need to expose patients to radiation. Discrepancies were noted in cases of mild pneumothorax or modest pleural effusion, without altering the clinical approach.

## 1. Introduction

Chest X-ray (CXR) is the most widely used methodic for the postoperative monitoring of patients after thoracic surgery procedures [1,2,3] for its availability, ease of use, and objectivity. However, it has inherent limitations: first of all, its diagnostic accuracy is lower than that of a CT scan; moreover, it exposes the patient to ionizing radiation, requires multiple healthcare personnel for execution and reporting, and is often not feasible shortly after surgery due to hospital organizational challenges [1,2,3,4,5].

Several studies have already advised against using CXRs as a means of patient monitoring after chest drain removal, claiming that clinical monitoring is sufficient in the vast majority of patients and restricting CXRs only to clinically symptomatic cases [6,7,8,9,10].

A lung ultrasound (LUS) can be rapidly performed at the patient’s bedside, can be repeated in cases of diagnostic uncertainty, and can be an accurate method for the planning of minimally invasive surgical interventions and for the prediction of pleural adhesions [11]. Its role in diagnosing respiratory issues has been well documented in emergency medicine contexts, but the literature regarding its routine use in evaluating postsurgical patients is limited. Moreover, it is already used in other surgical fields, like general surgery, as a rapidly feasible and repeatable diagnostic method at the bed of the patients. Many times, it is the only diagnostic method on which surgeons rely in order to make therapeutic decisions, such as the placement of a chest tube or the activation of an operating room [12,13].

The purpose of this study is to demonstrate the concordance of thoracic ultrasound compared to chest radiography, which currently represents the method of choice for the management of the postoperative period of patients after thoracic surgery. Once the concordance between the methods is proven, our results may be integrated into standardized protocols for the management of chest drainage, which could minimize the need for chest radiography.

## 2. Materials and Methods

During the period spanning from March 2022 to January 2024, we enrolled 280 adult patients who underwent thoracic surgery in the Thoracic Surgery Unit of Padua University Hospital. The inclusion criteria were as follows: age > 18 years and patients undergoing minor and major thoracic procedures (from nonanatomical lung resections to bilobectomy or thymectomy) via either a minimally invasive (VATS, video-assisted thoracoscopic surgery or RATS, robotic-assisted thoracoscopic surgery) or an open approach. Each patient gave written informed consent to participate. The exclusion criteria were pneumonectomy and the need for postoperative intensive care unit observation. All the patients in the postoperative course were followed-up with a chest X-ray, with the images evaluated by a surgeon. Simultaneously, a different surgeon blindly performed a bedside lung ultrasound, noting any anomalous finding. The pathological findings we were looking for included the following: absence of lung sliding, presence of lung point, absence of B lines, presence of “barcode sign” instead of “sand on the beach sign” on M-mode, and presence of pleural effusion, because, in our clinical practice, these are the common and clinically relevant outcomes in the postoperative setting. The same scheme was repeated in 219 out of the 280 patients after chest tube removal. The criteria used for chest tube removal in our unit were as follows: the absence of air leaks (or if an electronic pleural drainage was used, air leaks had to be between 0 and 20 mL/min) and liquid leaks under 200 mL/day of a nonhematic appearance. Some of the patients underwent a CXR between the two time points for the evaluation of proper lung expansion, but the investigators were blinded to the results.

The age, sex, presence or absence (and type) of pulmonary comorbidities, type of resection, and surgical approach were recorded.

Surgeons performed the LUS after training sessions with an expert ultrasound radiologist in order to acquire the appropriate ultrasound skills. An experienced interventional radiologist delivered theoretical lectures on thoracic ultrasound and its practical applications. An execution protocol was established to align with the needs of this study and the diagnostic findings to be investigated. Finally, each operator conducted practical exercises for approximately two weeks with experienced members of the Unit of Radiology staff.

The presence of pneumothorax on LUS examination was detected as follows: the operator slid the linear probe across the ribs for three to four intercostal spaces craniocaudally either in the parasternal, axillary, or posterior window, so that all the pathological chest cavity sections were thoroughly analyzed.

Meanwhile, the presence of pleural effusion on LUS examination was determined based on common clinical practice in the posterior paravertebral window.

The ultrasound and X-ray assessments were conducted at two time points:

1. Immediately after surgery (within a few hours after the surgical procedure).
2. The day following chest tube removal.

To standardize the procedure, the LUSs were performed for all patients in three areas homolateral to the surgical side (parasternal, mid-axillary, posterior paravertebral) in a supine or semi-seated position using the same ultrasound machine (TE7 Ace, Mindray Bio-Medical Electronics co.), using a linear probe (L12-3RCs with a frequency between 3.0 and 12.8 MHz) when evaluating pneumothorax and using a convex probe (C5-1S with a frequency between 1.0 and 5.0 MHz) in the evaluation of pleural effusion.

These findings were categorized as follows:

1. Negative.
2. Pneumothorax.
3. Pleural effusion.
4. Pneumothorax and pleural effusion.
5. Nondiagnostic.

For statistical purposes, a single summary result was assigned for the ultrasound, which was as follows:

1. “Negative”, if the ultrasound was negative.
2. “Positive”, if there was evidence of pneumothorax, effusion, or both in at least one area or nondiagnostic in all areas. A finding that is nondiagnostic means that either the patient was not compliant with cooperating with the US operator to change position for the examination or the postoperative dressing was too large to ensure an acceptable acoustic window. Another less common reason for a nondiagnostic LUS is the presence of a diffuse subcutaneous emphysema.

This dichotomization into two categories (positive and negative) was implemented for clinical purposes.

Similarly, the X-ray findings could be categorized as follows:

1. Negative.
2. Pneumothorax.
3. Pleural effusion.
4. Pneumothorax and pleural effusion.
5. Nondiagnostic. In this case, the CXR operator was not able to surely define the presence of a pneumothorax or an effusion due to the rehash of the pulmonary parenchyma.

A single summary result was then assigned, which was as follows:

1. “Negative”, if the X-ray was negative or nondiagnostic.
2. “Positive”, if the X-ray showed evidence of pneumothorax, effusion, or both.

### Statistical Method

The interobserver agreement between the two methods was determined using the Cohen’s kappa coefficient, both using dichotomous variables (positive and negative) and using the 5 categories of variables (LUS and X-ray findings). The sensitivity (SE), specificity (SP), and positive and negative predictive values (PPV, NPV) of the ultrasound were calculated based on the X-ray results using dichotomous variables (positive and negative).

The sample size was determined for an observational study comparing and analyzing the concordance between CXR and LUS methods. The procedure was based on calculating the sample size by defining a specific precision level for the estimation of Cohen’s kappa for the comparison between instrumental methods and a confidence level of 1 − α.

The sample size estimation was based on the following parameters:

1. Cohen’s kappa: 0.75.
2. Precision level (defined as the width of the confidence interval) for the estimation of Cohen’s kappa: 0.2.
3. Confidence interval for kappa: (0.65; 0.85).
4. Two raters (CXR and LUS methods).
5. Two categories (“positive” or “negative” for CXR and/or LUS).
6. Confidence level (1 − α): 95%.
7. Hypothesized proportions for the “positive”/“negative” categories: 0.25/0.75.

The estimated required sample size was 207 patients, as calculated using R software version 4.4.0 “presize” package. The statistical analysis was also performed using R software 4.4.0.

## 3. Results

The baseline population characteristics are reported in Table 1.

All the patients underwent ultrasound and X-ray examinations in the hours following their return from the operating room. Therefore, the results of 280 postoperative ultrasounds and 280 postoperative X-rays were collected. As reported in Table 2, when categorizing the results of these methods into “negative” and “positive” categories, the X-ray results were negative in 77.5% (217/280) and positive in 22.5% (63/280) of cases, while the ultrasound results were negative in 68.9% (193/280) and positive in 31.1% (87/280) of cases. Both methods concurred in evaluating patients as positive in 53 cases (84.1%) and negative in 183 (84.3%) cases. In 44 cases (31.6%), they disagreed: the ultrasound was negative in 10 cases that were positive on the X-ray and positive in 34 cases that were negative on the X-ray. The agreement rate was 84%, and the interobserver agreement, calculated using the kappa statistic, was 0.603 (*p*-value < 0.001).

The characteristics of the postoperative ultrasound, calculated in comparison to the X-ray as the reference method using dichotomous variables (negative and positive), were as follows (Table 3): sensitivity (SE) 84.1%, specificity (SP) 84.3%, positive predictive value (PPV) 60.9%, and negative predictive value (NPV) 94.8%. Performing the same analysis while excluding patients with comorbidities yielded overlapping results (agreement 82% with K statistic 0.558).

Out of the total 280 patients, 219 patients underwent an ultrasound examination after the removal of the drainage, as the portable ultrasound in the first year was not always available because it was shared with the pneumology unit.

By dividing the results of the methods into only two categories, “negative” and “positive”, the X-ray yielded a negative result in 66.7% of cases and a positive result in 33.3%, while the ultrasound yielded a negative result in 68% of cases and a positive result in 32%, as shown in Table 4.

Both methods concurred in evaluating patients as positive in 60 cases and negative in 136 cases. In 23 cases, they were discordant: the ultrasound was negative in 13 cases that were positive on the X-ray and positive in 10 cases that were negative on the X-ray. The agreement rate was 89%, and the interobserver agreement, calculated using the kappa statistic, was 0.761 (*p*-value < 0.001).

When the same analysis was performed excluding patients with comorbidities, an improvement in the kappa statistic was observed, resulting in k = 0.773, and the agreement rate was 90%.

Later, the results were also divided based on specific findings. The X-ray showed normal results in 66.7% (146/280) and detected pneumothorax in 14.2% (31/280), effusion in 13.2% (29/280), and both PNX and effusion in 5.5% (12/280). The ultrasound yielded normal results in 68% (149/280) and detected PNX in 13.7% (30/280), effusion in 14.6% (32/280), and both effusion and PNX in 2.3% (5/280). The agreement rate was 85%, and the kappa statistic was 0.702 (*p*-value < 0.001).

The characteristics of the drainage-removal ultrasound in comparison to the X-ray were as follows: sensitivity (SE) 82.2%, specificity (SP) 93.2%, positive predictive value (PPV) 85.7%, and negative predictive value (NPV) 91.3%, Table 5.

We decided to evaluate the possible improvement in the skills of the ultrasound operators over time by dividing the sample into two groups: patients examined in the first year (February 2022–January 2023) and those evaluated in the second year (February 2023–January 2024). Finally, the sample was also divided into patients with pulmonary comorbidities and patients without comorbidities to investigate whether the comorbidities had any influence on the level of agreement of the methods used. Separating the data collected in the first year of this study from those collected in the second year and calculating the concordance, a K statistic of 0.905 (vs. 0.687) was obtained for the drainage-removal assessment (agreement increased from 86% to 96%), while the K statistic was 0.839 (vs. 0.505) in the postsurgical setting (agreement increased from 79% to 95%).

In addition, between the first and second year, there was also a change in the sensitivity, specificity, PPV, and NPV of the positive and negative ultrasound findings compared with those of the X-ray, with a generic marked improvement, most evident in the postoperative phase (Table 6).

## 4. Discussion

Interpreting the values of Cohen’s kappa, there was a moderate agreement in identifying postoperative PNX and pleural effusion between the two methods used (k = 0.603). The level of agreement improved and was considered good when comparing the two methods at the time of drainage removal (k = 0.761). The sensitivity of the ultrasound was good, slightly higher in the postoperative phase (84.1%), while the specificity was higher after drainage removal (93.2%). Of great importance was the NPV (negative predictive value), which was 94.8% in the postoperative phase and 91.3% after drainage removal. This can be considered very satisfactory, especially when considering that it applies to effusions and pneumothoraces of any size, not just clinically significant ones. These values allow us to consider the ultrasound as a sufficiently safe method for ruling out PNX and pleural effusion.

**Figure 1:**
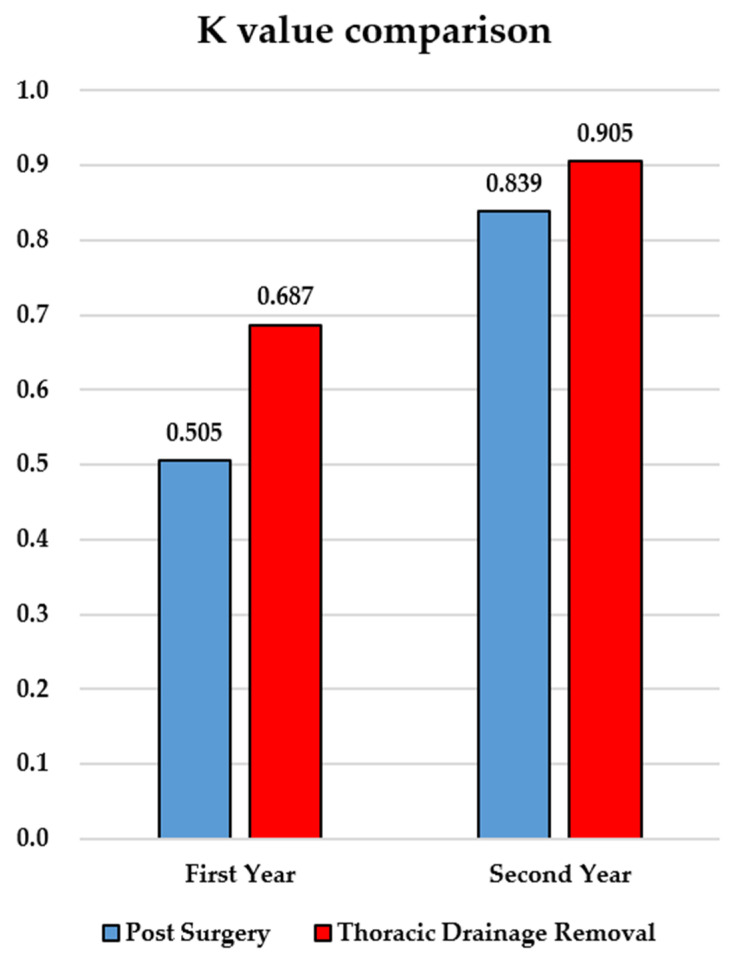
K-value comparison between LUS and CXR methods between postoperative and drainage-removal settings and between 1st and 2nd year of practice.

A limitation of all studies, including our own, that consider using ultrasound as a primary imaging technique in noncardiac thoracic surgery is the lack of a third method superior to ultrasound or X-ray as a comparison. Although CT (computed tomography) is superior to X-ray in detecting pneumothorax and effusion, it cannot be used in such studies because the patient would be exposed to significantly more ionizing radiation without any benefit. We are effectively comparing two imperfect imaging techniques, each with its own disadvantages and advantages. The lower agreement in the postoperative setting is likely due to various factors (Figure 1). Firstly, it is plausible that inflammatory changes in the lung parenchyma and pleura in the immediate postoperative setting may make it more challenging to detect and interpret ultrasound findings: recent surgical resection and pain may render difficult the proper, complete expansion of the pulmonary parenchyma, thus leading to a misdiagnosed pneumothorax; secondly, a small amount of flushing fluid and residual blood is always present in the pleural cavity after surgery, which may be interpreted as effusion. Moreover, the presence of slight subcutaneous emphysema in the surgical accesses may interfere with the proper evaluation of the underlying tissues. Also, in major demolitive interventions with ample mobilization of the lung parenchyma, pulmonary edema and contusions may alter the interpretation of findings in the US.

Secondly, postoperative pain makes it more difficult for the patient to cooperate. Specifically, during this study, we observed that patients, due to pain, were taking shallow breaths after leaving the operating room, making it harder to determine whether the absence of sliding was due to pneumothorax or to the patient’s limited ability to collaborate. In this context, it was more useful to rely on other diagnostic signs such as B-lines or by using the M-mode and evaluating the absence of the so-called “sand on the beach” sign. Additionally, detecting pleural effusion requires a seated position, which is difficult to maintain for patients in significant pain. It is possible that, at times, insufficient time was dedicated to performing the ultrasound in the posterior position.

About the disagreements between CXR and LUS, we observed that no different clinical approach would have been made if only one of the two was used. In fact, based only on the LUS findings, we noted that no patient who was discharged presented complications or needed another early hospitalization.

An advantage of the lung ultrasound may potentially concern economic savings, despite some preliminary considerations. A first economic and temporal investment is undoubtedly necessary for the training period of medical personnel carrying out the ultrasound imaging and for purchasing the ultrasound equipment (approximately EUR 20.000). Nevertheless, we noticed that, after about one year from the training, performing the bedside ultrasound would become a swift and well-standardized procedure, also allowing the achievement of a higher diagnostic yield. Considering the annual number of surgeries (approximately 800) and that the average cost of a chest X-ray is around EUR 25 at our hospital, the potential savings may be substantial in the long term.

From a recent systematic review written by Marek Malík et al. [9] about the comparison between lung ultrasound (LUS) and chest X-rays (CXRs) in postoperative care and chest tube management after noncardiac thoracic surgery, the percentage of agreement between them for pneumothorax varies from 72% to 92.3%, with a Cohen’s kappa from 0.397 to 0.775, and for pleural effusion, it varies from 38% to 88.1, with a Cohen’s kappa from 0.39 to 0.611. With these (and other, not reported) data, the authors conclude that “LUS as a primary imaging modality performed by an experienced thoracic surgeon combined with clinical examination can safely replace chest X-rays in decision making in postoperative care and chest tube management after noncardiac thoracic surgery without any negative impact on the patient’s outcome”. About the overall agreement, in the article from Ali Zein Elabdein et al. [14], a cross-sectional study, in which the operator performing the ultrasound examinations had completed a 6-month training period, was conducted on patients who underwent thoracic surgeries, and the authors found perfect overall agreement (K = 0.838, *p* < 0.001) between LUS and CXR in the postoperative period. In our study, the data show that, separating the data collected in the first year of this study from those collected in the second year and calculating the concordance, there was a clear increase, with a K statistic comparable to the previous example, equal to 0.905 for the drainage-removal assessment and to 0.839 in the postsurgical setting (perfect agreement). These findings could be useful in the elective surgical setting, while in other emergency settings, such as traumatized patients, ultrasound is the method of choice for the swift evaluation of pneumothorax [15].

In our experience, the lung ultrasound has proven to be a reliable and accurate method in the identification of pneumothorax and pleural effusion after the proper acquisition of the necessary skillsets. The concordance shown in this specific population suggests that the LUS may have important diagnostic value in patients who have undergone noncardiac thoracic surgery. However, it is necessary for these findings to be confirmed by further clinical studies, especially in order to establish a standardized protocol for performing and interpreting lung ultrasounds in a postsurgical context.

## 5. Conclusions

In conclusion, the LUS is a feasible and safe procedure for postoperative thoracic surgery patient evaluation. Despite its interobserver variability and the difficulty of performing it in certain conditions, the concordance between its findings and X-rays images is moderate or good depending on the context. The implementation of this method in algorithms for patient management by supplementing or replacing X-rays must be confirmed by further clinical studies.

## Figures and Tables

**Table 1:** Baseline patient characteristics.

Population Characteristics (*n* = 280)	
**Age (median, SD)**	62.45 (13.52)
**Gender**	
F	132 (47.1%)
M	148 (52.9%)
**Comorbidities**	
None	228 (81.4%)
Bronchial asthma	10 (3.6%)
COPD	9 (3.2%)
Interstitial lung disease	9 (3.2%)
Previous pneumonia	8 (2.9%)
Previous homolateral cancer	5 (1.8%)
Emphysema	1 (0.4%)
TBC	1 (0.4%)
Other	9 (3.2%)
**Disease**	
NSCLC	100 (35.7%)
Ground-glass opacity	74 (26.4%)
Lung metastasis	54 (19.35%)
Thymoma	10 (3.6%)
Pathology invading the chest wall	6 (2.1%)
Undetermined nature pleural effusion	5 (1.8%)
Recurrent pneumothorax	5 (1.8%)
Malignant pleural mesothelioma	3 (1.1%)
Other	23 (8.2%)
**Surgical Procedure**	
Lobectomy/bilobectomy	106 (37.9%)
Nonanatomical resection (wedge)	68 (24.3%)
Segmentectomy	51 (18.2%)
Thymectomy (with lung resection for tumor invasion)	10 (3.6%)
Diagnostic biopsy for persistent pleural effusion	8 (2.9%)
Sleeve lobectomy	5 (1.8%)
Pleurodesis for persistent pneumothorax (with bullae resection)	5 (1.8%)
Other	27 (9.6%)
**Surgical access**	
VATS	248 (88.6%)
Posterolateral thoracotomy	18 (6.4%)
Sternotomy	14 (5.0%)

COPD: chronic obstructive pulmonary disease; TBC: tuberculosis; NSCLC: non-small-cell lung cancer; VATS: video-assisted thoracoscopic surgery; SD: standard deviation.

**Table 2:** LUS and CXR pathological specific findings, postoperative.

	Postoperative X-ray	Postoperative LUS
Results		
Negative	217 (77.5%)	193 (68.9%)
Positive	63 (22.5%)	87 (31.1%)
**Agreement = 84.0%** **Kappa statistic = 0.603 (*p*-value < 0.001)**		
**Findings**		
Negative	217 (77.5%)	193 (68.9%)
PNX	47 (16.8%)	64 (22.9%)
Effusion	13 (4.6%)	14 (5.0%)
PNX + effusion	3 (1.1%)	3 (1.1%)
Nondiagnostic	0 (0.0%)	6 (2.1%)
**Agreement = 82.0%** **Kappa statistic = 0.580 (*p*-value < 0.001)**		

LUS: lung ultrasound; PNX: pneumothorax.

**Table 3:** LUS and CXR findings comparison, postoperative.

		Postoperative LUS		
		Positive	Negative	Total
**Postoperative CXR**	**Positive**	53 (84.1%)	10 (15.9%)	**63 (100.0%)**
	**Negative**	34 (15.7%)	183 (84.3%)	**217 (100.0%)**
	**Total**	**87 (31.1%)**	**193 (68.9%)**	**280 (100.0%)**

CXR: chest X-ray; LUS: lung ultrasound. In bold is reported the sum of positive and negative values (number and percentages).

**Table 4:** LUS and CXR pathological specific findings, drainage removal.

	Drainage-Removal X-ray	Drainage-Removal LUS
Results		
Negative	146 (66.7%)	149 (68.0%)
Positive	73 (33.3%)	70 (32.0%)
Agreement = 89.0% Kappa statistic = 0.761 (*p*-value < 0.001)		
Findings		
Negative	146 (66.7%)	149 (68.0%)
PNX	31 (14.2%)	30 (13.7%)
Effusion	29 (13.2%)	32 (14.6%)
PNX + effusion	12 (5.5%)	5 (2.3%)
Nondiagnostic	1 (0.5%)	3 (1.4%)
Agreement = 85.0% Kappa statistic = 0.702 (*p*-value < 0.001)		

PNX: pneumothorax. LUS: lung ultrasound.

**Table 5:** LUS and CXR findings comparison, drainage removal.

		Drainage-Removal LUS		
		Positive	Negative	Total
**Drainage-Removal CXR**	**Positive**	60 (82.2%)	13 (17.8%)	**73 (100.0%)**
	**Negative**	10 (6.8%)	136 (93.2%)	**146 (100.0%)**
	**Total**	**70 (32.0%)**	**149 (68.0%)**	**219 (100.0%)**

CXR: chest X-ray; LUS: lung ultrasound. In bold is reported the sum of positive and negative values (number and percentages).

**Table 6:** Difference between first- and second-year results.

First Year, Postoperative		Second Year, Postoperative	
Sensitivity	78.3%	Sensitivity	100%
Specificity	79.3%	Specificity	93.5%
Positive predictive value	55.4%	Positive predictive value	77.3%
Negative predictive value	91.7%	Negative predictive value	100%
**First Year, Chest Tube Removal**		**Second Year, Chest Tube Removal**	
Sensitivity	76.9%	Sensitivity	95.2%
Specificity	90.7%	Specificity	96.7%
Positive predictive value	83.3%	Positive predictive value	90.9%
Negative predictive value	86.7%	Negative predictive value	98.3%

## Data Availability

The data will be shared on reasonable request to the corresponding author.
